# Stable Radical Content and Anti-Radical Activity of Roasted Arabica Coffee: From In-Tact Bean to Coffee Brew

**DOI:** 10.1371/journal.pone.0122834

**Published:** 2015-04-09

**Authors:** Gordon J. Troup, Luciano Navarini, Furio Suggi Liverani, Simon C. Drew

**Affiliations:** 1 School of Physics, Monash University, Victoria, 3800, Australia; 2 illycaffé Spa, via Flavia 110, 34147, Trieste, Italy; 3 Florey Department of Neuroscience and Mental Health, The University of Melbourne, Victoria, 3010, Australia; Martin-Luther-Universität Halle-Wittenberg, GERMANY

## Abstract

The roasting of coffee beans generates stable radicals within melanoidins produced by non-enzymatic browning. Roasting coffee beans has further been suggested to increase the antioxidant (AO) capacity of coffee brews. Herein, we have characterized the radical content and AO capacity of brews prepared from *Coffea arabica* beans sourced directly from an industrial roasting plant. In-tact beans exhibited electron paramagnetic resonance signals arising from Fe^3+^, Mn^2+^ and at least three distinct stable radicals as a function of roasting time, whose intensity changed upon grinding and ageing. In coffee brews, the roasting-induced radicals were harboured within the high molecular weight (> 3 kD) melanoidin-containing fraction at a concentration of 15 nM and was associated with aromatic groups within the melanoidins. The low molecular weight (< 3 kD) fraction exhibited the highest AO capacity using DPPH as an oxidant. The AO activity was not mediated by the stable radicals or by metal complexes within the brew. While other non-AO functions of the roasting-induced radical and metal complexes may be possible *in vivo*, we confirm that the *in vitro* antiradical activity of brewed coffee is dominated by low molecular weight phenolic compounds.

## Introduction

Coffee brews contain many polyphenols, well known for their antioxidant (AO) properties [1]. During roasting of coffee beans, endogenous AOs in green beans, such as chlorogenic acids, are partly decomposed or incorporate into melanoidins [2]. The latter are a heterogeneous group of polymers with variable molecular weights that derive from the Maillard reaction between reducing sugars and amino acids during roasting (non-enzymatic browning) [3]. Although there is substantial evidence that the biological activity of coffee melanoidins and flavonoids *in vivo* may derive from non-AO mediated mechanisms [4–9], numerous studies have focused upon the AO activity of coffee brews and extracts [2,7,10–16].

It is well established that roasting of coffee beans produces stable radicals [17–21], which are associated with Maillard reaction products (MRPs) [2] rather than phenolic substituents [12]. Bekedam et al showed that the formation of non-phenolic roasting-induced AOs positively correlated with melanoidin content within coffee brew, but that these roasting-induced AOs are slow-reacting and have a limited contribution to the overall AO activity in comparison with phenolic constituents [2]. The role of the roasting-induced radical within coffee brews has yet to be explicitly considered.

Although the radical content of roasted coffee has been studied under laboratory conditions that emulate industrial roasting processes [20], a controlled, time-dependent study utilizing beans sourced directly from an industrial roaster is yet to be investigated. We examined *Coffea arabica* (Arabica) beans from an industrial roasting plant in order to measure the stable radical content of in-tact and ground beans as a function of roasting time and the relationship between the radical content and AO activity in coffee brewed from these beans. Using electron paramagnetic resonance (EPR), we identified at least three different stable radical species as a function of roasting times ranging from 2–12 min, each of which shows enhanced intensity upon grinding, with further increases upon ageing of the grounds. We suggest an enhancement with grinding and ageing may result from the exposure of a greater surface area and/or gradual dehydration, both of which will result in an increased microwave penetration depth. In coffee solutions brewed using dark-roasted beans, we demonstrate that the radical(s) are localized predominantly within high-molecular-weight phenolic-containing melanoidins and have no oxidant or antioxidant properties in an *in vitro* antiradical assay.

## Materials and Methods

### Origin and Preparation of Samples

*Coffea arabica beans* (wet processed, screen size ≥15, zero primary and secondary defects, high aromatic profile, clean cup) were sourced from Brazil and roasted at temperature up to 220°C under industrial conditions for durations of 2, 4, 6, 8, 10 and 12 minutes. Samples (25 g for each roasting time) were immediately sealed under inert atmosphere in individual foil bags prior to shipping and further analysis. Australian quarantine restrictions prevented us from importing unprocessed green coffee beans. Upon receipt, the foil bags were opened and the beans were studied either in-tact or following grinding. Coarse-ground samples were prepared by crushing ca. 10–20 beans with a marble mortar and pestle. To assist grinding, 2 min roasted beans were first frozen in liquid nitrogen.

To prepare coffee brews, 3.5 g of 12 min-roasted beans was coarse ground. Within 15 min of grinding, 20 mL ‘MilliQ’ grade water (Millipore) was added and brewed at 92 ± 1°C in a beaker on a heat block with constant stirring. After 5 min, the solutions were rapidly cooled on ice and clarified using a low-binding Millex 0.45 μm PVDF syringe filter (Millipore). The resulting solutions were either used immediately or stored at—80°C prior to further use.

### Removal of phenolics from coffee brew

To determine the association of phenolics with radical content and antiradical activity, coffee brew was treated with polyvinylpolypyrrolidone (PVPP; Fluka), which adsorbs phenolics [22]. The brew was first diluted in MilliQ water by a factor of 62.5 (in preparation for antiradical assay; *vide infra*) and treated with 100 mg/mL PVPP for 90 min at room temperature on a rotary mixer. The slurry was then briefly centrifuged at low speed to sediment most of the water-insoluble PVPP, before a final pass through a 0.45 μm PVDF syringe filter.

### Removal of metal ions from coffee brew

To chelate metal ions, a 100 mM solution of diethylenetriaminepentaacetic acid (DTPA; Sigma-Aldrich) was prepared by dissolution in NaOH. To 500 μL of coffee brew, 5 μL of 100 mM DTPA (“+DTPA” condition) or 5 μL MilliQ water (“–DTPA” condition) was added. The pH of the brew changed only slightly (from pH 5.3 to 5.5) in the presence of the alkaline DTPA solution. For each treatment condition, the resulting solution was transferred to an Amicon Ultra 3kD centrifugal ultrafiltration device containing a low-binding regenerated cellulose membrane (Millipore), centrifuged at 20,200 g for 10 min and the filtrate collected. An additional 450 μL MilliQ water was added to the retentate and the solution centrifuged for a further 10 min. This procedure was repeated once more before finally returning the high molecular weight (HMW) species within the retentate to their original concentration by addition of MilliQ water to a final volume of 500 μL. The proportion of low molecular weight (LMW) species remaining in the retentate after the serial dilutions was estimated to be 0.1–0.5%.

### EPR spectroscopy

Continuous-wave EPR spectra were acquired at 25°C using an E500 X-band spectrometer (Bruker) fitted with a Bruker super-high-Q probehead (ER 4122SHQE). Coarse-ground beans were contained in 3 mm ID quartz sample tubes (Wilmad, 707-SQ). No attempt was made to correct for the different packing density of grounds prepared for different roasting conditions. Whole beans were inserted directly into the probehead by attaching them to the end of a 3 mm ID quartz EPR tube using paraffin film (Bemis Company, Inc.). Six beans from each roasting condition were first weighed on a microbalance, then individually inserted into the center of the microwave probehead with the “face” of the bean being approximately parallel to the direction of the static magnetic field. No attempt was made to correct for difference in mass (S2 Fig), cavity filling factor or the lower microwave penetration depth of lighter-roasted beans arising from their higher water content. Solution measurements of coffee brews were made under the same conditions as above, except that samples were contained in a quartz flat cell (Wilmad, WG-808-Q).

### EPR spectral decompositions

Spectra corresponding to individual radical species were isolated by weighted subtraction of spectra of whole/ground beans obtained at short, medium and long roasting times, followed by normalisation of the resultant spectrum by double-integration. Using the basis spectra so obtained, all experimental spectra were then empirically decomposed by adding linear combinations of normalized basis spectra and adjusting relative weightings to minimise the difference between the empirical reconstruction and experiment. Fitting was guided by minimisation of the mean-squared deviation between the reconstruction and the experimental spectrum and the requirement for smooth and continuous changes in composition as a function of roasting time.

### EPR spectral simulations

Tumbling-averaged (brew) and randomly oriented (ground coffee and whole bean samples) EPR spectra were simulated using version 1.1.4 of the XSophe-Sophe-XeprView computer simulation software [23]. Where *g* factors are quoted in the text, they have been corrected using a solution spectrum of DPPH in 1:1 MeOH/water as a magnetic field calibration standard (*g* = 2.0036).

### DPPH antiradical assay

A 100 μM solution of 2,2-diphenyl-1-picrylhydrazyl (DPPH; Sigma-Aldrich) was prepared in methanol. Coffee solutions were serially diluted in MilliQ water by factors of 62.5, 125, 250,…, 8000, 16000. To each well of a 96-well clear microplate (Greiner), 100 μL of each dilution was added in duplicate or triplicate, then 100 μL of 100μM DPPH solution was added to all wells to initiate the reaction. After 60 min incubation at 25°C, the absorbance at 517 nm was read using a SPECTROstar Nano (BMG LABETCH) spectrophotometer. The small intrinsic absorbance of brewed coffee at 517 nm was corrected for at the highest concentrations tested. The solute concentration that reduced the initial DPPH• concentration by 50% (EC50) was computed from the dose response curve by non-linear least squares fitting to the 4-parameter sigmoidal equation [24]:

$$y=top-bottom+\frac{top-bottom}{1+{\left[\frac{\text{EC}50}{x}\right]}^{slope}}$$ (1)

where *x* is the compound concentration after mixing with DPPH, ‘*slope*’ is the Hill slope (corresponding to the steepness of the curve), *y* is the DPPH^•^ concentration, ‘*top*’ is the maximum response (constrained to be >0.95) and ‘*bottom*’ is the baseline response (constrained to be <0.05). Curve fitting and calculation of the mean and standard error in the parameters was performed using GraphPad Prism 5 software using the data from three repeats of the plate assay, with each repeat using a brew prepared on a separate occasion. No precipitation of any component was observed within the time frame of the assay.

### Statistical analyses

Statistical analyses were carried out using GraphPad Prism 5 software. The relevant statistical test applied is indicated in the figure legends. Graphs show the mean and standard error of the mean (SEM) of ‘n’ independent experiments unless otherwise stated.

## Results

### EPR spectroscopy of coffee beans

**Fig 1:**
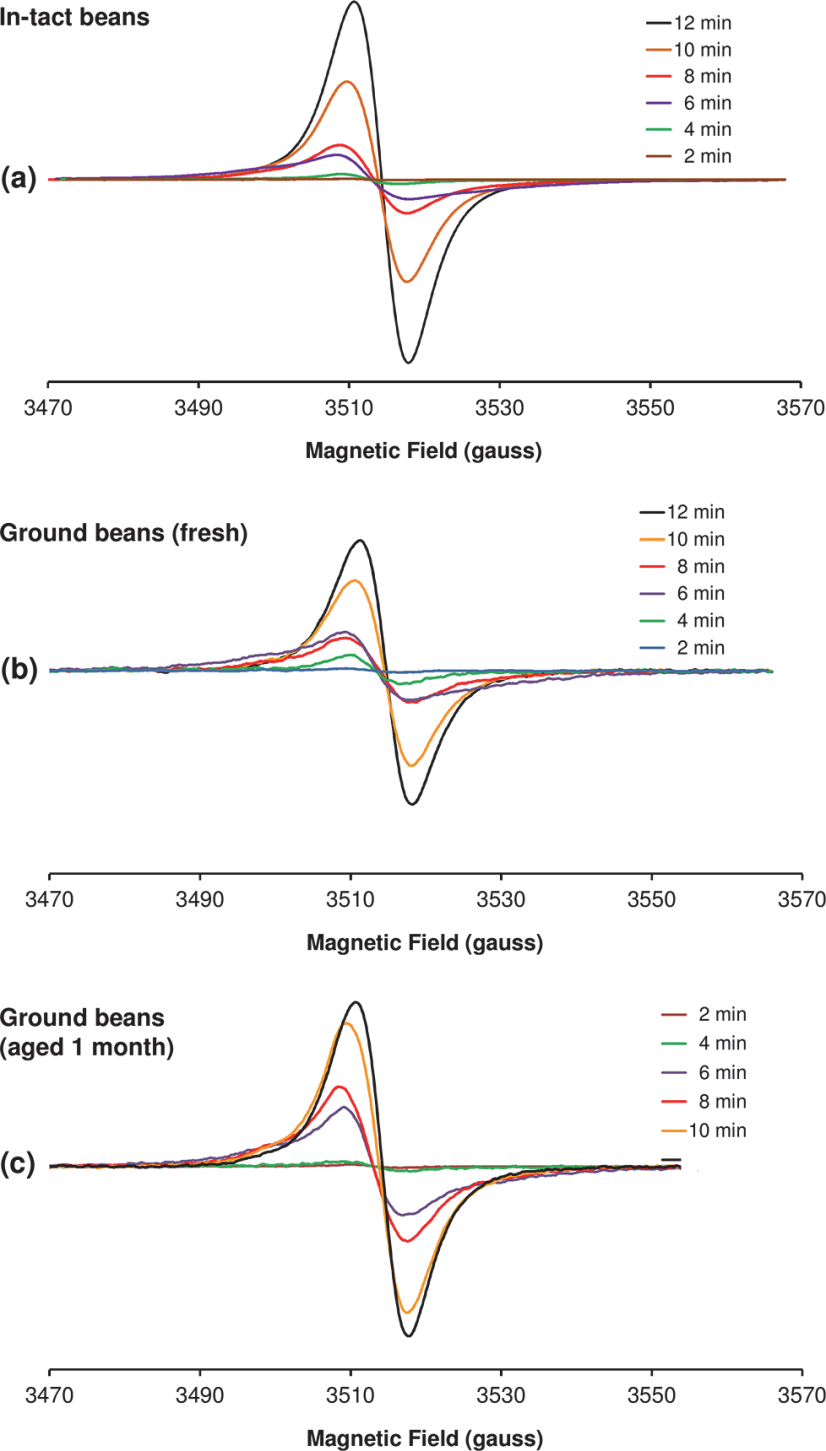
Dependence of the radical EPR spectrum on roasting time in (a) in-tact beans and (b) freshly-ground beans and (c) the same grounds aged 1 month. Data in *a* represents the average of the double-integrated intensity of EPR spectra obtained from 6 individual beans (S3 Fig) at each roasting time. Experimental conditions: microwave frequency, 9.860 GHz; microwave power, 0.1 mW; magnetic field modulation amplitude, (*a*) 3 G, (*b*,*c*) 1 G; field modulation frequency, 100 kHz; receiver time constant, 164 ms; receiver gain, (*a*) 60 dB, (*b*,*c*) 66 dB; sweep rate, 4 G/s; averages, 1. The intensity in *b-c* is directly comparable, whereas the intensity in *a* is not directly comparable with *b-c*.

**Fig 2:**
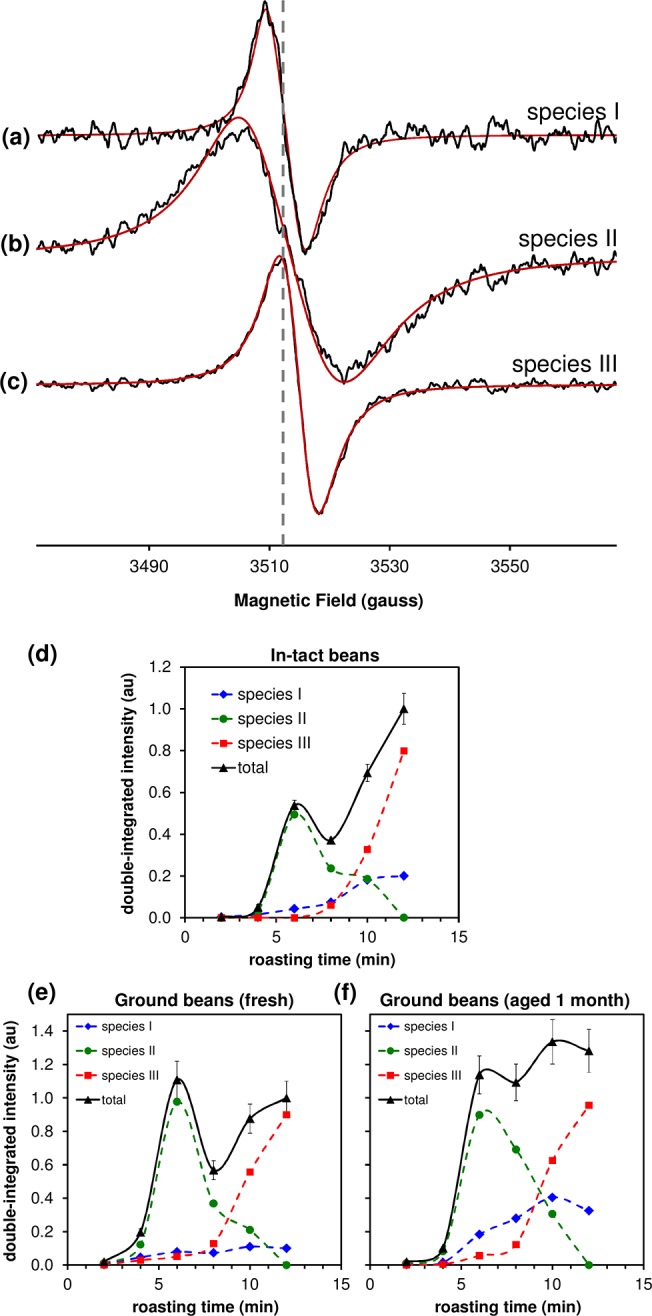
Multiple radical species are identified during roasting. **(a–c)** Experimental (―) and simulated (―) EPR spectra of three putative radicals present in whole and ground coffee beans, isolated as shown in S4 Fig Using the basis spectra in *a–c*, the total EPR signal intensity (determined by double integration) of the experimental spectra from **(d)** in-tact beans, **(e)** freshly ground and **(f)** same grounds aged 1 month, was decomposed into contributions from species I–III. Details of the decompositions are shown in S5–S7 Figs The dashed vertical line in *a*–*d* is positioned at the line center of species I to aid comparison of the different spectra. Error bars in *d* represent the mean and standard error of the intensity of the EPR spectra from individual beans. Error bars in *e*-*f* depict an arbitrary ±10% error associated with EPR spectra from a single sample of ground beans. Note that the intensity in *e-f* is comparable, but the intensity in *d* is not directly comparable with *e-f*.

In-tact coffee beans exhibit EPR spectra comprising broad, anisotropic signals characteristic of high spin Fe^3+^, Mn^2+^ and narrow signal(s) corresponding to a stable radical(s) [25] (see also S1 Fig). Fig 1 shows the EPR spectra of the stable radical in whole coffee beans and in freshly-ground beans that were roasted for variable times ranging from 2–12 min. At maximum roasting (12 min), the spectrum was characterized by a *g* factor of ≈ 2.004 and a linewidth of 7 G, consistent with the radical species previously observed in roasted coffee [20] and instant coffee [12,26], in addition to several other beverages and foodstuffs including caramelized glucose [12]. Quantification of the radical intensity (determined by double-integration of the first derivative signal) showed a monotonic increase as a function of roasting time except for a local maximum after 6 min roasting, which was associated with the presence of a broad component underlying the more narrow signals present at short and long roasting times (Fig 2). Through spectral algebra and simulations (S3–S6 Figs), we were able to isolate and characterize three separate radical species (I–III) as a function of roasting time. The distribution curves for in-tact and ground beans are shown in Fig 2. Whilst the basis spectra (S4 Fig) chosen are not necessarily unique, at least three such spectra were required in order to satisfactorily decompose the experimental data in Fig 2.

Although the temperature and time dependence of the roasting procedure differed, Goodman *et al*. observed a similar dependence of the integrated EPR intensity during the roasting of *Robusta* and *Arabica* half-beans directly within the variable-temperature gas flow accessory of the EPR spectrometer (cf. their Fig 1 and our Fig 2) [27]. Moreover, they also observed a broad EPR signal at intermediate roasting times that is comparable with species II. They determined that the broad EPR signal must correspond to an unstable radical formed during roasting because its intensity rapidly declined with additional roasting [27]. In contrast, we conclude that our species II must correspond to a stable radical, since in our case the spectroscopy was carried out long after roasting. This suggests that the radical(s) produced at intermediate roasting times are stable under ambient conditions but are perhaps subsequently deteriorated by further roasting.

The relative enhancement of the EPR intensity of species II upon grinding could suggest the broad radical signal originates from air exposure of a roasting-induced radical. An enhancement of the double-integrated EPR intensity upon cutting or grinding of whole beans has been previously observed by Yeretzian *et al*. [21], although the experimental EPR spectra upon such treatment were not shown. Paramagnetic oxygen is well known to broaden radical EPR spectra by an exchange broadening mechanism [28–29]; indeed, it was possible to simulate species II using an isotropic *g* factor and a lineshape with mixed Lorentzian (80%) and gaussian (20%) character, which might be consistent with oxygen broadening of the radical. However, while this might account for the larger linewidth of the radicals produced at intermediate roasting times, it does not explain the relative enhancement of species II intensity immediately following air exposure.

One possible explanation is that species II is not oxygen sensitive *per se* but rather belongs to radicals that reside predominantly within the interior of medium-roasted beans, which may be less accessible at X-band due the their higher water content (and thus increased dielectric loss and reduced microwave penetration depth). These radicals may then be detected in greater number upon grinding due to exposure of radicals previously inaccessible to the microwave field within the resonator and may continue to grow in apparent intensity due to enhanced rate of dehydration of the grounds with age. In this instance, the large linewidth might arise from the presence of a heterogeneous distribution of radical sites in chemically distinct environments that exist during intermediate stages of melanoidin formation. This interpretation would be consistent with both a higher residual linewidth and “*g* strain” parameter for species II as compared with the other radical spectra identified (Table 1).

**Table 1:** Spin Hamiltonian parameters determined from numerical simulation of the experimental EPR spectra.

	***g***_||_	***g***_⊥_	⟨***g***⟩ ^*a*^	σ_R||_ ^*b*^	σ_R⊥_ ^*b*^	σ(***g***_||_)/***g***_||_ ^*c*^	σ(***g***_⊥_)/***g***_⊥_ ^*c*^
Species I	2.0067	2.0038	2.0048	0.5	2.63	0.00060	0.00039
Species II ^*d*^	2.0085	2.0017	2.0045	2.07	4.38	0.00177	0.00231
Species III	2.0050	2.0030	2.0039	1.62	2.52	0.00082	0.00068
Brew ^*e*^	–	–	2.0039	2.98	2.98	–	–

Uncertainty in *g* estimated ±0.0001.

^*a*^ ⟨*g*⟩ = (*g*_||_ + 2*g*_⊥_)/3.

^*b*^ Lorentzian residual linewidth (×10^–4^cm^–1^).

^*c*^ *σ* = width of gaussian distribution of *g* values (“*g* strain”).

^*d*^ This broad spectrum could also be simulated using an isotropic *g* factor of 2.0047 and a phenomenological isotropic linewidth of ≈80% Gaussian and 20% lorentzian character.

^*e*^ Solution spectrum of brew prepared from 12 min-roasted beans.

Species I is present at low roasting times, suggesting this signal may correspond to endogenous radicals present within the green bean. The signal intensity appears to increase with roasting time, although some of the increase may once again be due to the enhanced microwave penetration depth into the bean upon the gradual dehydration during roasting (*vide supra*). Species III predominates at longer roasting times and grows rapidly at roasting times > 8 min, suggesting it is associated with roasting-induced radicals formed during the latter stages of the Maillard reaction. The EPR signal associated with species III is narrower than species II, which concomitantly decreases, suggesting the radical products formed during the latter stages of melanoidin formation converge to a less heterogeneous population.

Ageing of the ground beans for 1 month within the unsealed EPR sample tube led to additional changes in the distribution of species I–III (cf. Fig 2E and 2F). In particular, the total radical signal detected was increased at roasting times > 6 min. Once again, this observation might be correlated with a gradual decrease in water content during exposure of the grounds to air, leading to improved microwave penetration depth and number of spins detected.

### EPR spectroscopy of brewed coffee solutions

**Fig 3:**
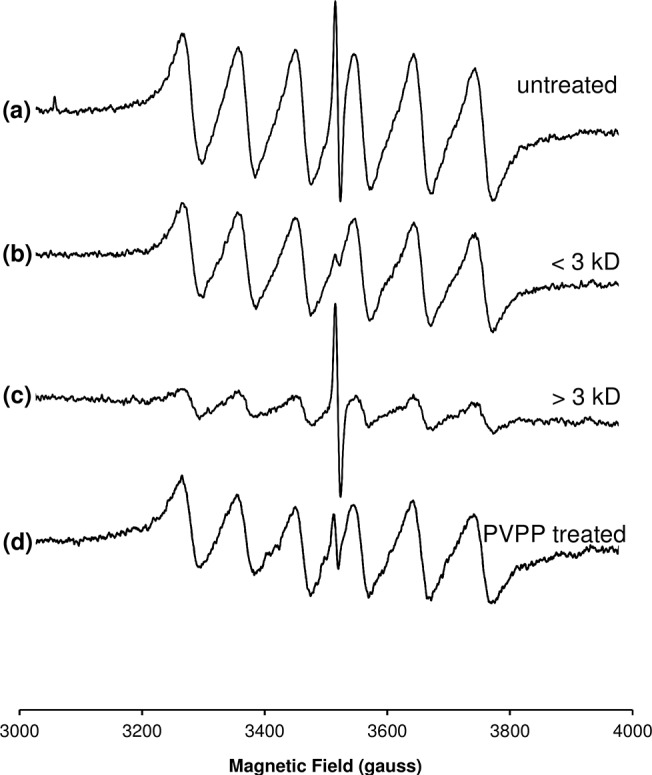
Room temperature EPR spectra of coffee brew following various treatments. **(a)** untreated brew; **(b)** filtrate from the first pass through the 3K MWCO membrane, representing the species < 3kD at their approximate native concentration; **(c)** retentate after three passes through the 3K MWCO membrane and reconstituted to the initial volume of brew prior to filtration, representing species > 3kD at their native concentration; **(d)** unfiltered brew treated with PVPP as described in the methods. Results are representative of a single brew. Microwave frequency 9.860 GHz microwave power, 50 mW; modulation amplitude, 10 G; receiver time constant, 327 ms; receiver gain, 82 dB; sweep rate, 6.67 G/s; number of averages, 4.

The room-temperature EPR spectrum of coffee brewed from dark (12 min) roasted beans is simpler than those corresponding to whole coffee beans due to the averaging of anisotropic interactions in tumbling solution. The spectrum consisted of a sextet of lines of width 25–30 G and spaced by 90–100 G arising from interaction of the electron spin with a ^55^Mn^2+^ nucleus (I = 5/2), together with a single-line radical species of width ~5 G (Fig 3A). The isotropic ⟨*g*⟩ = 2.0039 of the radical extracted by hot water was consistent with that of the predominant species (III) in the ground 12 min-roasted beans (Table 1, S8 Fig). The narrower linewidth in solution as compared with the solid state suggests a small degree of *g* anisotropy and heterogeneity (“*g* strain”), which is consistent with the spectral simulations (Table 1). By comparison with the EPR spectrum of a [Mn(H_2_O)_6_]^2+^ standard solution, the concentration of Mn^2+^ in the brew was determined (by double-integration of the spectra) to be ≈13 μM. Similarly, the concentration of the stable radical in the brew was estimated to be 15 nM by comparison with a standard sample of DPPH in solution.

Melanoidins have been proposed to impart AO activity through an ability to chelate transition metal ions [9]. Non-SOD Mn-based AOs are known to be present in complex biological systems [30] and have also been synthesized [31]. The catechol group of flavonoids provides a metal chelating functionality [5] and a variety of nitrogen and oxygen ligands within of melanoidins provide additional metal chelating groups [9]. Given a Mn^2+^ signal was observed in the EPR spectra of both in-tact beans and coffee brews, we therefore determined whether Mn^2+^ was likely bound to melanoidins or existed as LMW species. Fig 3 shows the EPR spectra of LMW (3 kD) and HMW (>3 kD) fractions of brewed coffee solution. From the intensity of the Mn^2+^ spectra, one may estimate that ≈75% of the Mn^2+^ is contained within the LMW fraction.

**Fig 4:**
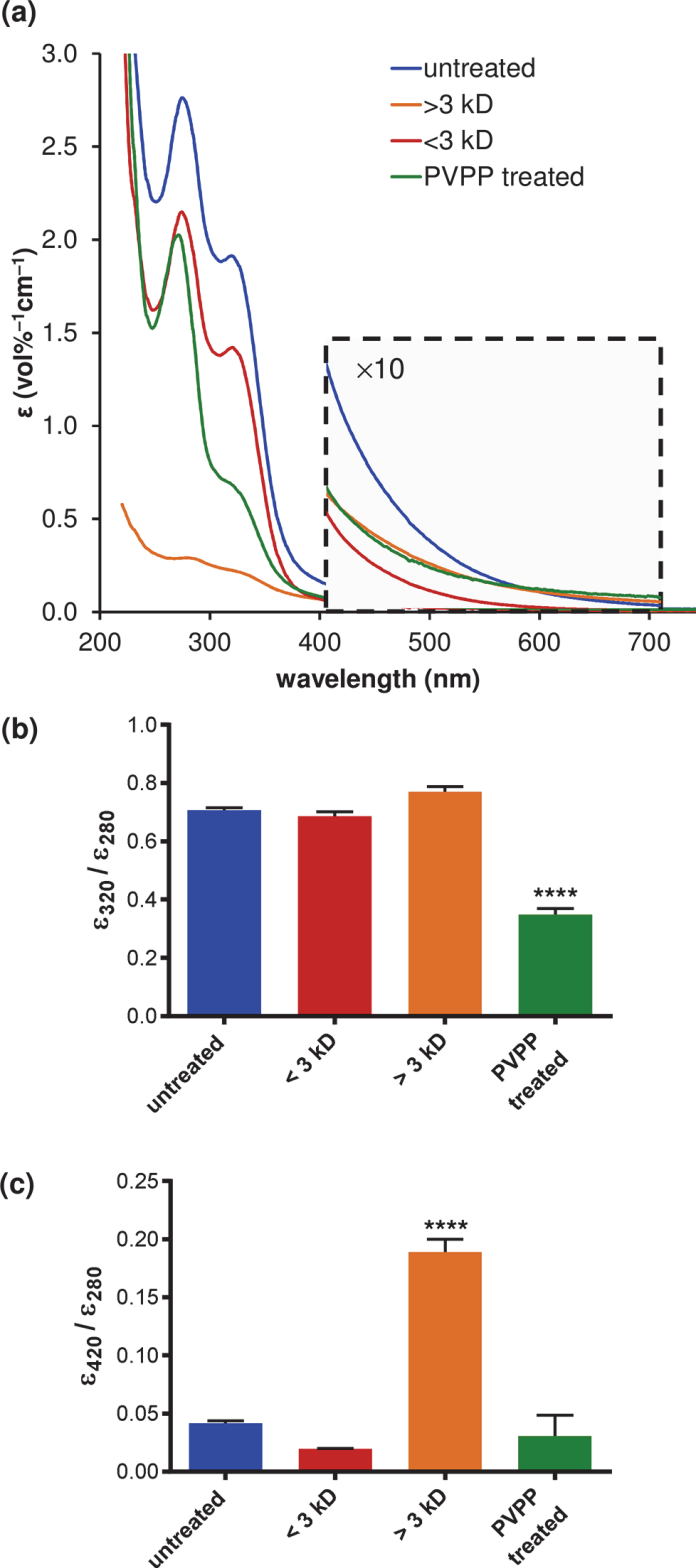
(a) UV-vis spectra of coffee brew following various treatments; quantification at discrete wavelengths associated with (b) total protein and polyphenol content (280 nm, 320 nm) and (c) melanoidins (420 nm). Consistent with a reduction in chlorogenic acid content [3], a significant decrease in the relative absorbance at 320 nm versus 280 nm was observed following phenolic extraction with PVPP, (one-way ANOVA, *F* = 24.77, *P* < 0.0001, *n* = 3; Holm-Šídák test, ****P < 0.0001 vs. untreated, < 3 kD and > 3 kD). A significant increase in the relative absorbance at 420nm versus 280 nm was also observed in the HMW fraction following 3 kD filtration (one-way ANOVA, *F* = 57.91, *P* < 0.0001, *n* = 3; Holm-Šídák test, ****P < 0.0001 vs. untreated, < 3 kD, PVPP treated), in keeping with brown color intensity arising from HMW melanoidins.

In contrast with Mn^2+^ content, the radical signal was more intense in the HMW fraction (Fig 3). The radical signal was better resolved by pre-treatment of the brew with DTPA, which chelates a vast array of metal ions with high affinity [32], but whose Mn(DTPA) EPR spectrum is undetectable at mildy acidic pH (S9 Fig) [33]. We estimated that >95% of the stable radical is contained within the HMW fraction, indicating that the radical resides within the melanoidin structure. The presence of melanoidins (and caramelization products) in the HMW fraction was confirmed by the higher color intensity of this fraction at 420 nm (Fig 4). Caramelization produces species with a molecular weight range (less than 2 kD to over 10 kD) [34] that overlaps with that of melanoidins [4,9] and is known to produce stable EPR-detectable radicals with *g* values comparable with those in coffee and other beverages [12]. Previous EPR studies have indicated that the stable radicals in soluble coffee remain detectable following removal of phenolics by PVPP [12]; however, we observed that PVPP treatment markedly reduced the radical concentration within the coffee brew (Fig 3). We therefore ascertained that the radical species resides within aromatic groups within the melanoidin. It is currently unclear whether these aromatics derive from protein side chains or incorporated phenolics such as chlorogenic acid.

### Antiradical activity of brewed coffee solutions

**Fig 5:**
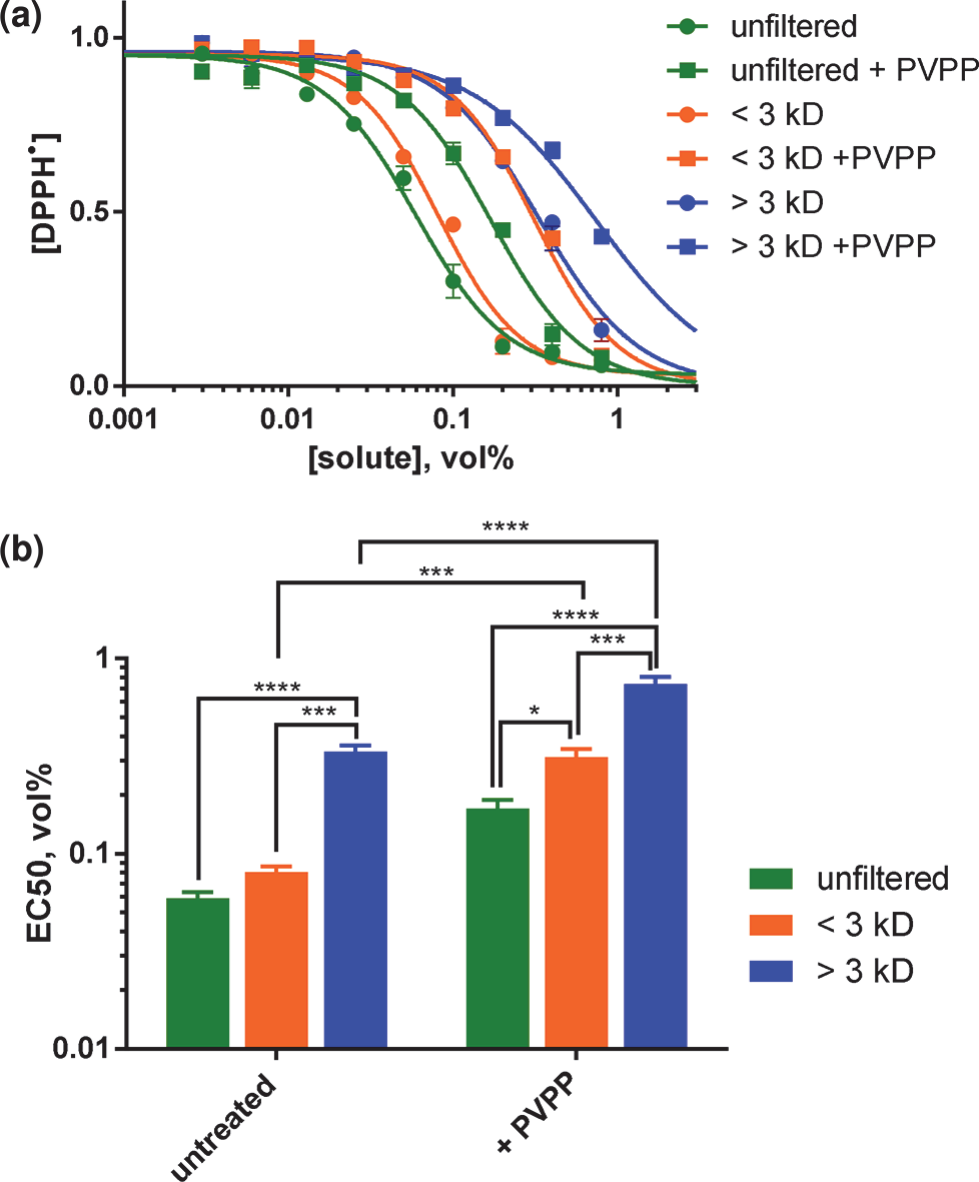
Reduction of DPPH^•^ to its hydrazine form DPPH-H in the presence of brewed coffee. (a) Dose response curves; (b) EC50 values calculated from the curves. The brew was optionally pre-treated with PVPP, followed by filtration through a 3 kD MWCO membrane. Reactions contained MeOH/water (1:1) solutions of 50 μM DPPH and varying dilutions of coffee brew. Significant differences were calculated by two-way ANOVA using the Holm-Šídák multiple comparison test (*n* = 3): ****P < 0.0001, ***P < 0.001, **P < 0.01, *P < 0.05. Numerical EC50 data is provided in S1 Table.

Fig 5 shows the dose response curves for the antiradical activity of brewed coffee in 1:1 MeOH/water and of coffee solutions subjected to various treatments. The EC50 of the retentate was more than 5-fold higher than the unfiltered brew, demonstrating that the highest antiradical activity is contained within the LMW fraction (< 3kD). Removal of phenolics by PVPP treatment increased the EC50 of the filtrate four-fold (S1 Table), indicating that a significant fraction of the antiradical activity of the LMW fraction can be attributed to phenolic-containing species. Since ≈60% of coffee melanoidins are contained within the 12–15 kD molecular weight range [9], these results suggest that LMW species containing aromatic functional groups are the major contributors to antiradical activity of the brew. Finally, treatment with DTPA, a high affinity chelator of metal ions including Mn^2+^, caused no significant change to the retentate EC50 (S10 Fig and S1 Table), indicating that metal binding to coffee melanoidins or flavonoids, particularly Mn^2+^, does not contribute to the antiradical activity of brewed coffee. For comparative purposes, we also performed the DPPH assay using Trolox under identical solution conditions (1:1 MeOH/water, 50 μM DPPH). The EC50 was computed to be 11.3 ± 1.6 μM (S11 Fig).

**Fig 6:**
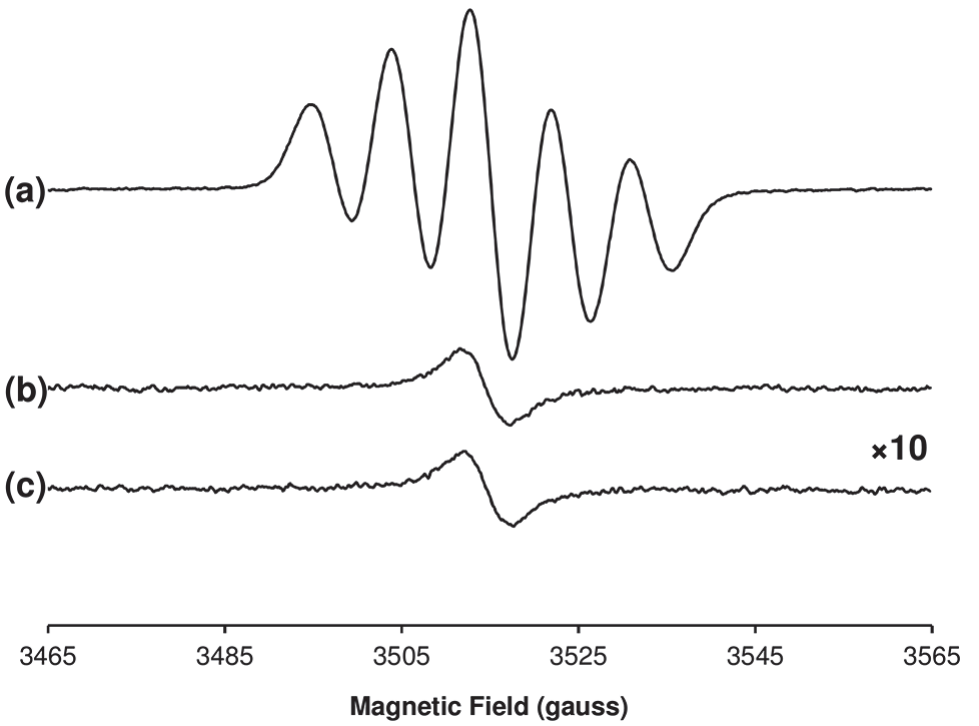
Antiradical activity of brewed coffee does not consume or generate stable coffee radicals. Room-temperature CW-EPR spectra of 1:1 MeOH/water solutions containing (a) 50 μM DPPH^•^ alone, (b) brewed coffee added to an equal volume of 100 μM DPPH^•^ in MeOH, and (c) brewed coffee added to an equal volume of MeOH. Data represent results of assay performed on a single brew. For clarity, the vertical scale is an order of magnitude higher spectra in *b* and *c* as compared with *a*. Experimental conditions: microwave frequency, 9.860 GHz; microwave power, 10 mW; magnetic field modulation amplitude, 4 G; field modulation frequency, 100 kHz; receiver time constant, 82 ms; receiver gain, 70 dB; sweep rate, 4 G/s; averages, (*a*) 4, (*b*,*c*) 40.

We also examined the behavior of the stable radical during the DPPH assay using EPR spectroscopy, which confirmed the direct reduction of DPPH• (characterized by a 5-line EPR spectrum) to non-paramagnetic DPPH-H in a dose-dependent manner (S12 Fig) comparable with the UV-vis spectrophotometric assay. Adding a neat brewed coffee solution to an equal volume of 100 μM DPPH• completely quenched the 5-line EPR signal, but the intensity of the stable coffee radical was unaffected (Fig 6). By comparing the double-integrated intensity of the radical spectra of DPPH and the coffee brew, the stable radical concentration within the brew was estimated to be 30 nM. Being three orders of magnitude lower than the DPPH• concentration, the stable coffee radical cannot be associated with any significant antiradical activity. This is in line with our observation that the radical is contained within the HMW fraction of the brew, which contains the lowest antiradical activity (Fig 3). Our finding is also consistent with a study of soluble coffee, where a stable coffee radical was still present even after the coffee solution had quenched a DEPMPO-OH• radical adduct [26]. Finally, it is clear from Fig 6 that the antiradical activity of the brew does not generate any other stable radicals as end-products.

## Discussion

The chemical rationale for the AO activity of coffee brews is complex. A number of polyphenols are present in green coffee beans and during roasting these polyphenols are converted into a plethora of compounds (mostly by transformation, degradation and polymerization). At the same time, the Maillard reaction and caramelization (another form of non-enzymatic browning) occur, generating a number of compounds characterized by a wide range of molecular weights.

The determination of the relative AO activity of MRPs and polyphenols to the AO activity of coffee has frequently focused upon chemical extraction from roasted beans or lyophilized brew, with activities being normalized by weight of each fraction. However, their relative AO activity when present at their normal concentration in the brewed beverage is of more relevance to dietary AO intake through coffee consumption. We observed that the highest antiradical activity was associated with the LMW fraction (<3 kD) of the brew, suggesting a predominant role of polyphenols (non-bound to melanoidins). In support of this, extraction of phenolics by PVPP treatment reduced the EC50 of the brew to a level comparable with that exhibited by the HMW fraction (Fig 5). This is consistent with the linear correlation between phenolic (gallic acid) content and AO activity observed by Brezová et al for ground and soluble coffee [11], and the finding of Bekedam *et al*. that phenolic AOs dominate the overall AO activity of coffee brews despite melanoidins possessing measurable AO activity [2].

Phenolic AOs within the brew react exceedingly slowly or not at all with the roasting-induced radical species observed by EPR, since the paramagnetic signal in Fig 5 is extremely stable. Non-paramagnetic antioxidants (A) within the brew can react with DPPH^•^ via both radical and non-radical pathways [35]. Where unstable radical intermediates (A^•^) are formed, these may undergo subsequent homolytic addition reactions with other DPPH^•^ molecules [35] or other AO radical intermediates to form non-paramagnetic (EPR-silent) derivatives. Similarly, the stable coffee radical (R^•^) detected by EPR (Fig 4) may effect an AO activity of its own, by undergoing homolytic addition reactions with AO intermediates A^•^ and/or DPPH^•^. Indeed the antiradical activity of hot water extracts increases as a function of roasting time of the beans [13], and the stable radical within melanoidins could contribute to the observed roasting-induced increase in AO activity of melanoidins. However, it is clear from Fig 6 that the radical species present in the brew maintains a constant intensity in the presence of DPPH. Moreover, no evidence for formation of new stable radical species was observed, indicating the EPR-detectable stable radical in the brewed coffee possesses no significant AO activity as measured by the DPPH assay. The lack of DPPH reactivity does not preclude an AO role for the roasting-induced radical against other oxidants, or when other phenolic-based AO species have been exhausted. Moreover, other non-AO mediated roles for the roasting-induced radical cannot be precluded *in vivo*, including cell signaling events. However, with a radical concentration of only 15 nM and an EC50 of 0.059 vol% in the presence of 50 μM DPPH^•^ (S1 Table), one can estimate that the roasting-induced radical is at least six orders of magnitude lower in concentration than the AOs responsible for the antiradical activity.

Despite the potential for both flavonoids [5] and melanoidins [9] to chelate transition metals, the presence of non-SOD Mn-based AOs in complex biological systems [30] and the strong AO activity of some LMW Mn^2+^ complexes [31], we did not observe any significant change in DPPH antiradical activity upon Mn^2+^ chelation with DTPA. This finding is consistent with similar studies on a range of wines, where no significant correlation between the Mn^2+^ content and AO activity could be discerned [36]. However, while such metal complexes may not show significant DPPH antiradical activity, our results do not preclude other AO mechanisms of metal-polyphenol complexes through inhibition of Fenton chemistry [37].

Consistent with previous analyses of soluble coffee [12], treatment with PVPP to remove phenolics decreased the color of the brew. Moreover, the HMW fraction was darker than the LMW fraction following 3 kD filtration (and reconstitution). Thus, phenolics may be bound to the color-generating centers within the melanoidins (and caramelization) species at HMW. However, the lower antiradical activity of the HMW fraction indicates that bound phenolics are not present at large enough concentration to provide melanoidins with a higher AO activity than the unbound phenolics within the LMW fraction. The lower antiradical activity of the darker HMW fraction is also consistent with previous analyses of amino acid–glucose model systems, which revealed no correlation between antiradical activity and color index of melanoidins [4].

In summary, this study applied EPR spectroscopy and *in vitro* antiradical assays to study the radical content and antiradical capacity of *Coffea arabica* sourced from an industrial roasting plant. The present research demonstrated that a number of stable radical species are formed during roasting and their intensity profile varies with roasting time and upon subsequent grinding and ageing. The stable radical(s) present within dark-roasted beans are unrelated to the antiradical activity of coffee brewed from those beans; however, this does not preclude a functional role for these radical species in non-antioxidant mechanisms *in vivo* following coffee consumption, or in variations to flavor profile during storage and ageing. A greater understanding of the origin and fate of roasting-induced radicals might further be obtained by employing EPR imaging techniques to enable the correlation of the radical species identified herein with their spatial location within the in-tact bean.

## Supporting Information

S1 Fig Representative room-temperature EPR spectra of an in-tact coffee bean roasted for 12 min. The experimental parameters were optimal for the transition metals, such that the radical spectrum is “clipped”. The Mn^2+^ may contribute additional structure underlying the low field features ascribed to Fe^3+^ [25]. Experimental conditions: microwave frequency, 9.860 GHz; microwave power, (*a*) 50 mW, (*b*) 20 mW; magnetic field modulation amplitude, 8 G; field modulation frequency, 100 kHz; receiver time constant, 82 ms; receiver gain, 76 dB; sweep rate, (*a*) 10 G/s (*b*) 6.7 G/s; averages, (*a*) 1 (*b*) 2.
(TIFF)

S2 Fig Average mass of coffee beans as a function of roasting time. Data represents the mean and SEM from measurement of 6 individual beans at each roasting time. The beans are the same as those used to generate the EPR spectra in Fig 1A (main text).
(TIFF)

S3 Fig Double-integrated EPR intensity of 6 randomly selected beans contributing to the average data presented in Fig 1 (main text). The average intensity of the 2 min roasted beans is one order of magnitude lower than the 4 min roasted beans and cannot be viewed on the vertical scale of the plot.
(TIFF)

S4 Fig Isolation of three putative radical species from the EPR spectra of in-tact coffee beans. (a) species I; (b) species II; (c) species III. Simulated spectra are shown overlaid in red and the corresponding simulation parameters appear in Table 1 (main text).
(TIFF)

S5 Fig Decomposition of the EPR spectra of *in-tact* coffee beans using the basis spectra shown in Fig 2. The experimental spectrum is shown in black and the reconstituted spectrum (weighted summation of species I–III) is shown in orange. The weightings are shown as a function of roasting time in Fig **2**D (main text). The vertical scale of displayed spectra is arbitrary in each instance.
(TIFF)

S6 Fig Decomposition of the EPR spectra of *freshly-ground* coffee beans using the basis spectra shown in Fig 2. The experimental spectrum is shown in black and the reconstituted spectrum (weighted summation of species I–III) is shown in orange. The weightings are shown as a function of roasting time in Fig **2**E (main text). The vertical scale of displayed spectra is arbitrary in each instance.
(TIFF)

S7 Fig Decomposition of the EPR spectra of *ground* coffee beans (*aged 1 month*) using the basis spectra shown in Fig 2. The experimental spectrum is shown in black and the reconstituted spectrum (weighted summation of species I–III) is shown in orange. The weightings are shown as a function of roasting time in Fig **2**F (main text). The vertical scale of displayed spectra is arbitrary in each instance.
(TIFF)

S8 Fig Simulation of the room-temperature EPR spectrum of brewed coffee. **(a)** Wide scan of the Mn^2+^ and radical signals. Microwave frequency, 9.866 GHz microwave power, 5 mW; modulation amplitude, 4 G; receiver time constant, 82 ms; receiver gain, 85 dB; sweep rate, 6.67 G/s; averages, 250. **(b)** Narrow scan of the radical signal, with a simulation overlaid in red. Microwave power, 10 mW; modulation amplitude, 4 G; sweep rate, 4 G/s; averages, 40. Simulation parameters are given in Table 1 in the main text.
(TIFF)

S9 Fig Room temperature EPR spectra of low (< 3 kDa) and high (> 3 kDa) molecular weight fractions of brewed coffee in the absence (––––) and presence (––––) of the metal chelator DTPA (1 mM). Due to its short electron spin lattice relaxation time, the Mn(DTPA) complex is effectively EPR-silent at room temperature. Hence, the complete sequestration of Mn^2+^ from the brew (eg. from complexes with melanoidins, polyphenols or from adventitious ions in solution) was confirmed by the disappearance of the six-line signature. **(a)** untreated brew; **(b**) filtrate from the first pass through the 3K MWCO membrane, representing the species < 3 kDa at their native concentration; **(c)** retentate reconstituted to the initial volume of brew, representing species > 3 kD at their native concentration. Results are representative of a single preparation of 12 min roasted beans brewed at 0.175 g/mL for 5 min at 92°C. Microwave frequency 9.860 GHz microwave power, 50 mW; modulation amplitude, 10 G; receiver time constant, 327 ms; receiver gain, 82 dB; sweep rate, 6.67 G/s; number of averages, 4.
(TIFF)

S10 Fig Dose response curves for the reduction of DPPH^•^ in the presence of coffee solutions containing 1mM DTPA. The EC50 does not change, indicating coffee metal-polyphenol and metal-melanoidin complexes do not contribute to antiradical activity.
(TIFF)

S11 Fig Dose response curve for the reduction of DPPH^•^ by Trolox in 1:1 MeOH/water. Data show the mean and SEM of 4 independent experiments.
(TIFF)

S12 Fig Reduction of DPPH^•^ to its hydrazine form DPPH-H in the presence of brewed coffee monitored by EPR. Room-temperature CW-EPR spectra of 1:1 MeOH/water solutions containing **(a)** 50 μM DPPH^•^ alone, or 50 μM DPPH^•^ with brewed coffee diluted in water by a factor of **(b)** 32000, **(c)** 8000, **(d)** 4000, **(e)** 3000, **(f)** 2000, **(g)** 1500, **(h)** 1000, or **(i)** 500. **(j)** Intensity of the DPPH^•^ EPR signal shown in spectra *b–i*, normalized to the intensity of the 50 μM DPPH solution in spectrum *a*. Data represent results of a single assay performed on one brew and are therefore only qualitative. Experimental conditions: microwave frequency, 9.866 GHz; microwave power, 10 mW; magnetic field modulation amplitude, 4 G; field modulation frequency, 100 kHz; receiver time constant, 82 ms; receiver gain, 70 dB; sweep rate, 4 G/s; averages, 4.
(TIFF)

S1 Table Numerical EC50 values corresponding to the graphical data in Fig 5 (main text). Data represent the mean and SEM obtained from three brews prepared and assayed independently.
(DOCX)
