# Supplementary figures and images for: Stable Radical Content and Anti-Radical Activity of Roasted Arabica Coffee: From In-Tact Bean to Coffee Brew
Source: PLoS One. 2015 Apr 9;10(4):e0122834. doi: 10.1371/journal.pone.0122834 (PMC4391752; doi:10.1371/journal.pone.0122834)

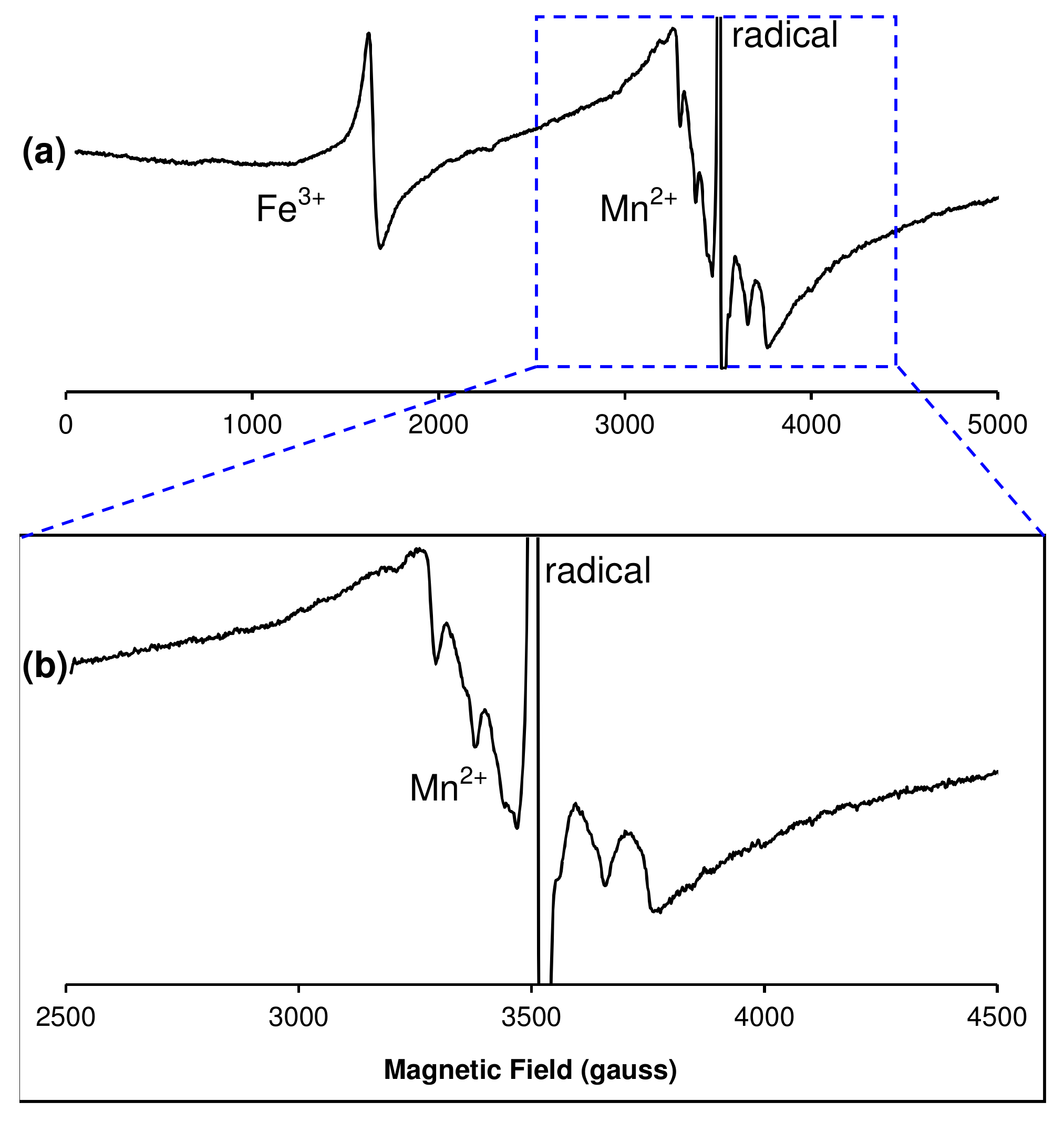

Supplement: S1 Fig — The experimental parameters were optimal for the transition metals, such that the radical spectrum is “clipped”. The Mn2+ may contribute additional structure underlying the low field features ascribed to Fe3+ [25]. Experimental conditions: microwave frequency, 9.860 GHz; microwave power, (a) 50 mW, (b) 20 mW; magnetic field modulation amplitude, 8 G; field modulation frequency, 100 kHz; receiver time constant, 82 ms; receiver gain, 76 dB; sweep rate, (a) 10 G/s (b) 6.7 G/s; averages, (a) 1 (b) 2. (TIFF) [file pone.0122834.s001.tiff]

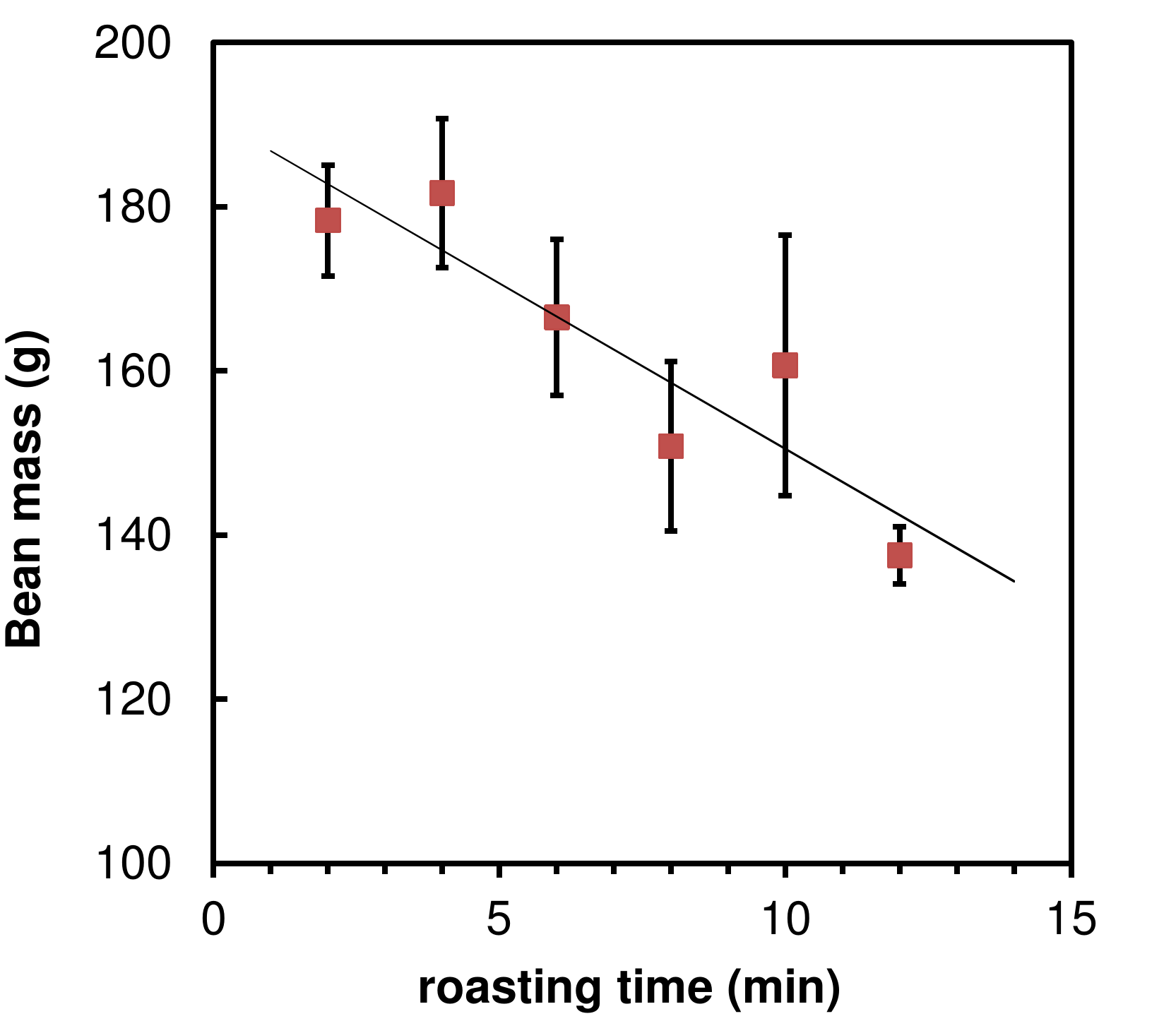

Supplement: S2 Fig — Data represents the mean and SEM from measurement of 6 individual beans at each roasting time. The beans are the same as those used to generate the EPR spectra in Fig 1A (main text). (TIFF) [file pone.0122834.s002.tiff]

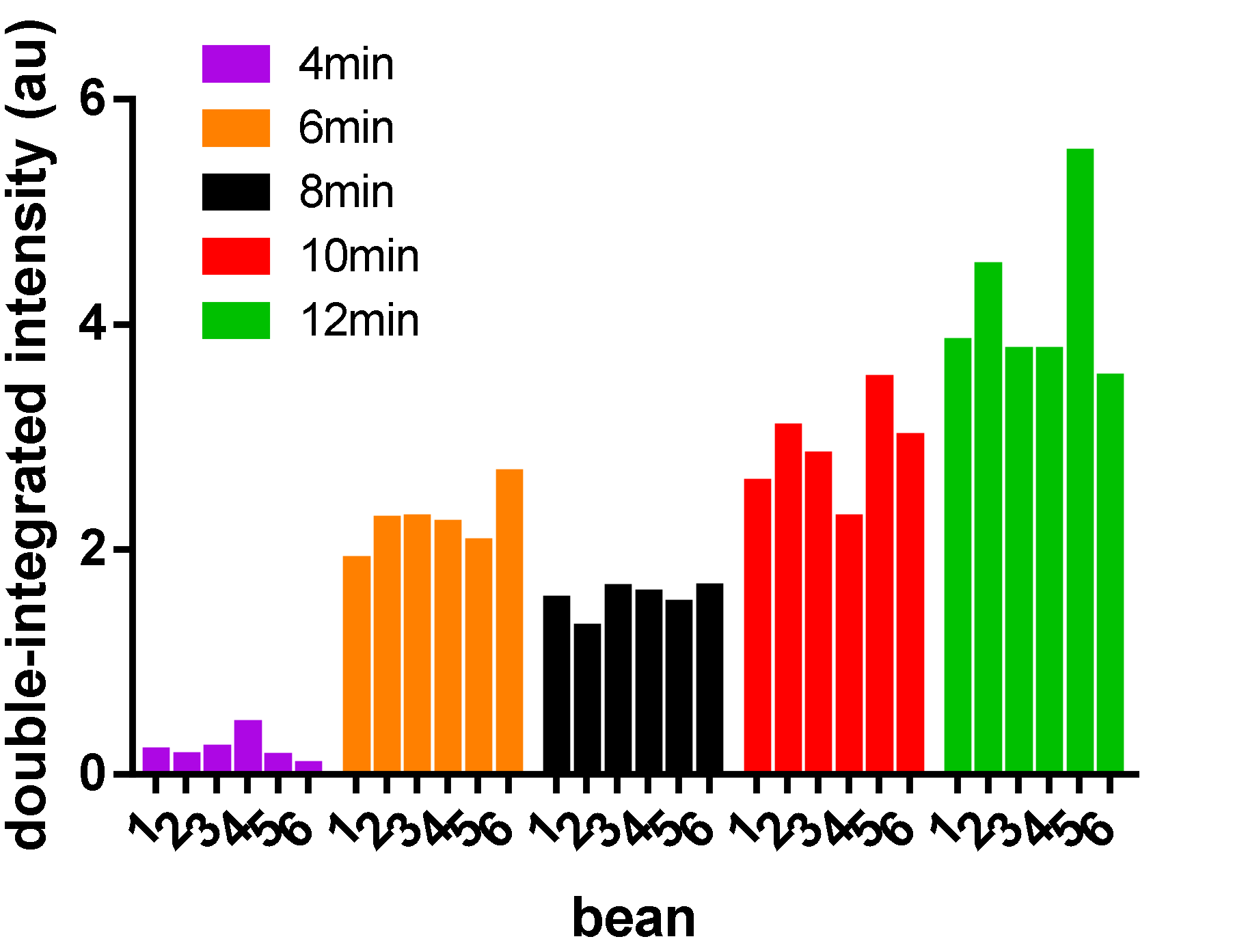

Supplement: S3 Fig — The average intensity of the 2 min roasted beans is one order of magnitude lower than the 4 min roasted beans and cannot be viewed on the vertical scale of the plot. (TIFF) [file pone.0122834.s003.tiff]

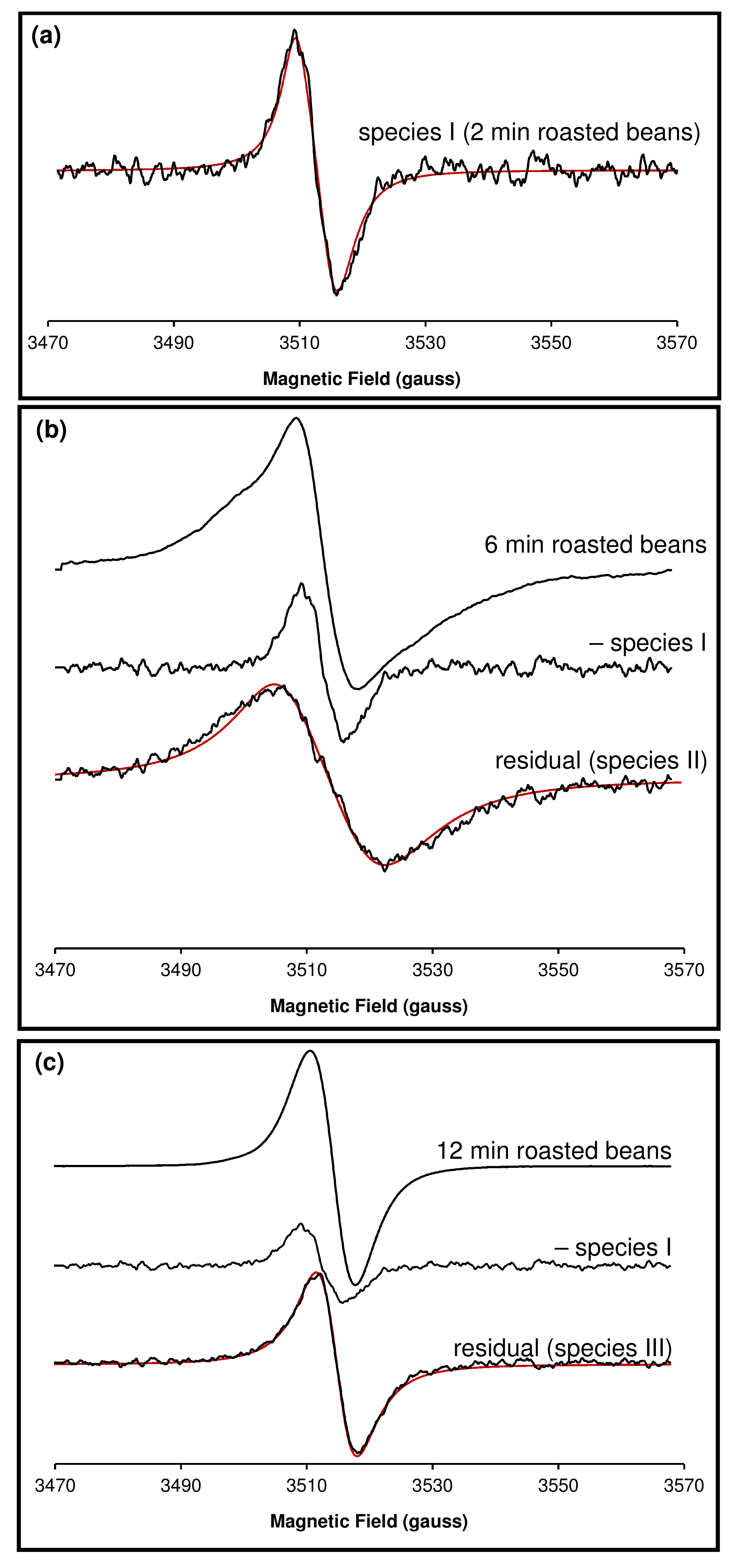

Supplement: S4 Fig — (a) species I; (b) species II; (c) species III. Simulated spectra are shown overlaid in red and the corresponding simulation parameters appear in Table 1 (main text). (TIFF) [file pone.0122834.s004.tiff]

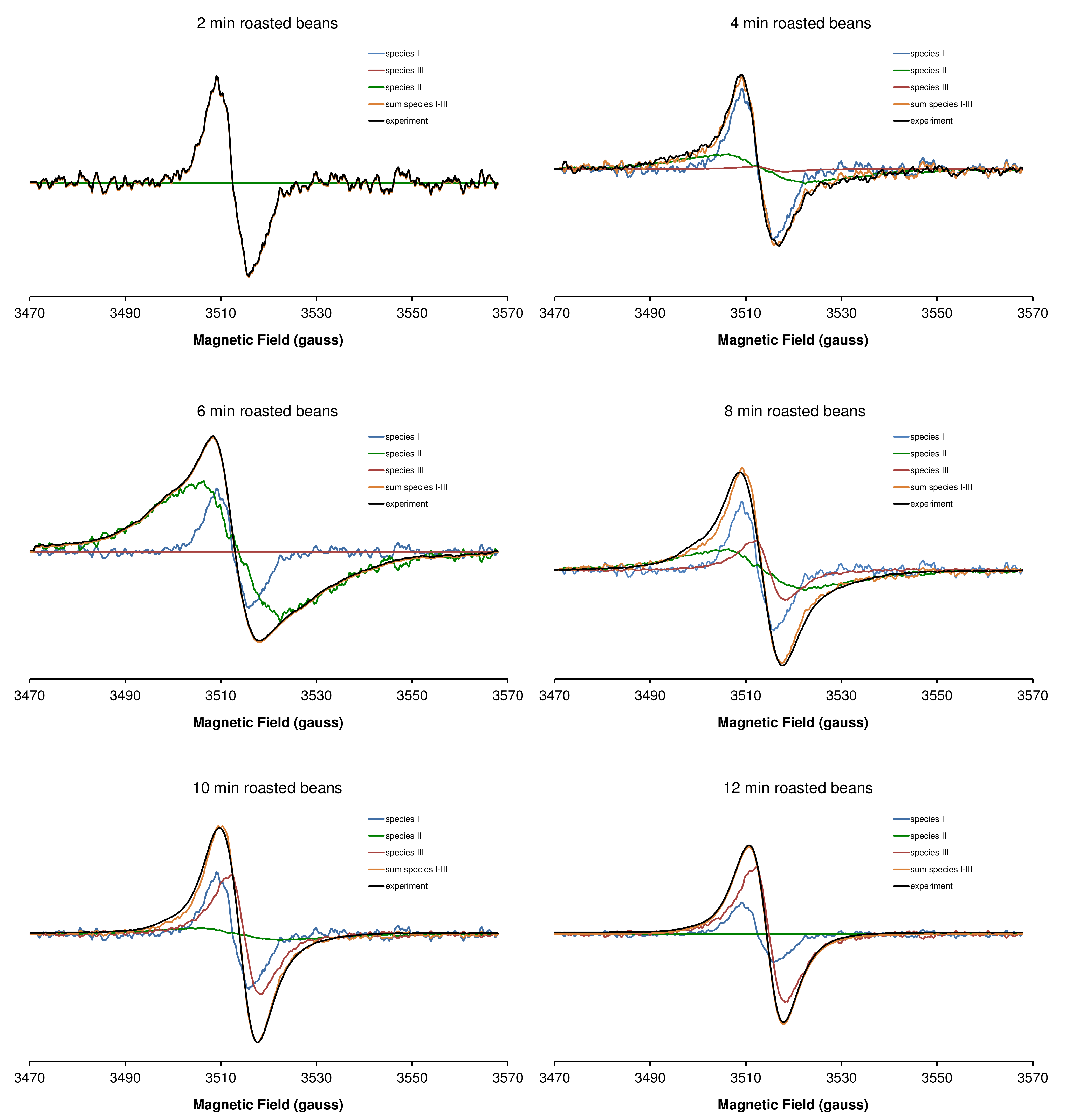

Supplement: S5 Fig — The experimental spectrum is shown in black and the reconstituted spectrum (weighted summation of species I–III) is shown in orange. The weightings are shown as a function of roasting time in Fig 2D (main text). The vertical scale of displayed spectra is arbitrary in each instance. (TIFF) [file pone.0122834.s005.tiff]

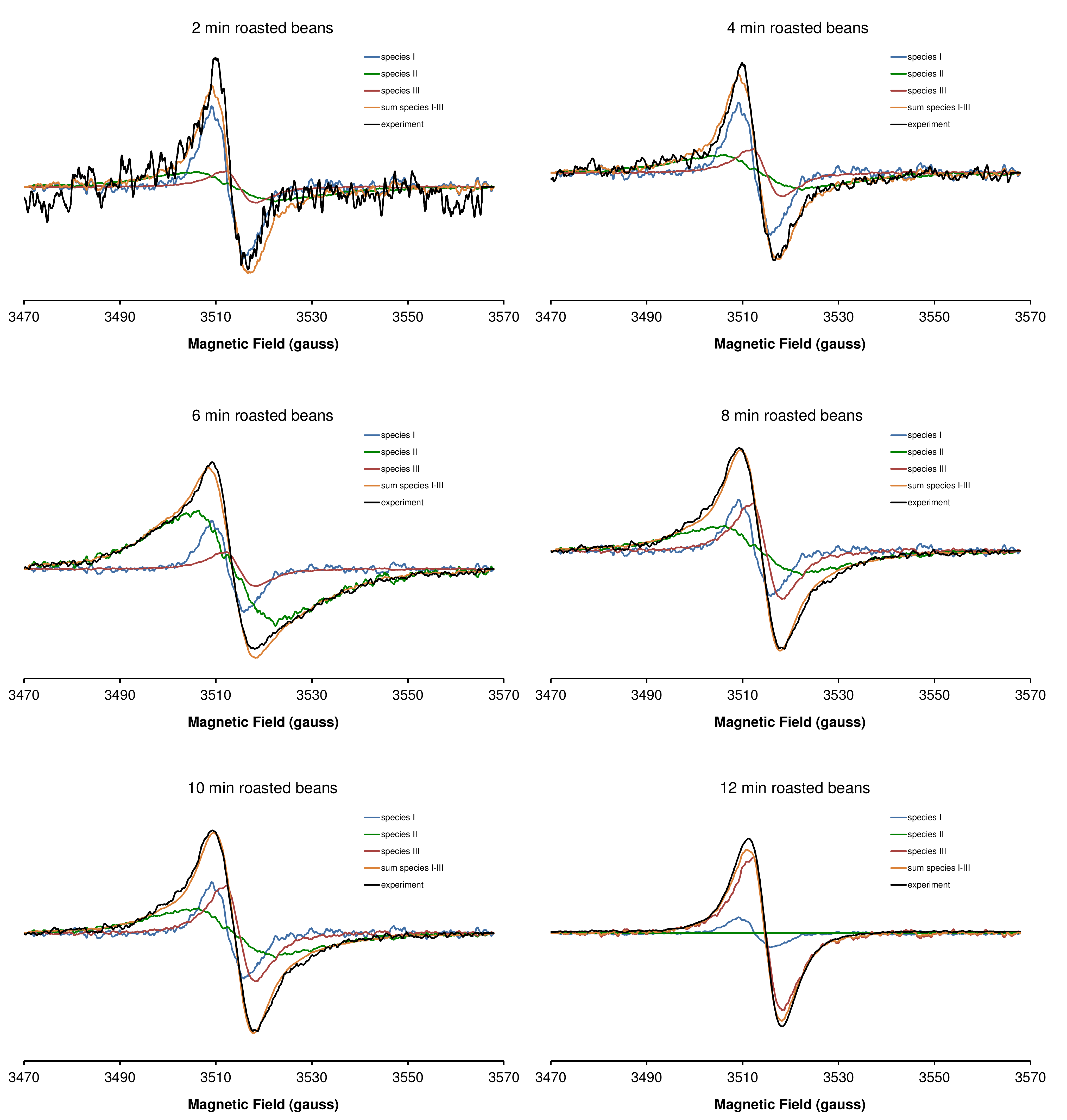

Supplement: S6 Fig — The experimental spectrum is shown in black and the reconstituted spectrum (weighted summation of species I–III) is shown in orange. The weightings are shown as a function of roasting time in Fig 2E (main text). The vertical scale of displayed spectra is arbitrary in each instance. (TIFF) [file pone.0122834.s006.tiff]

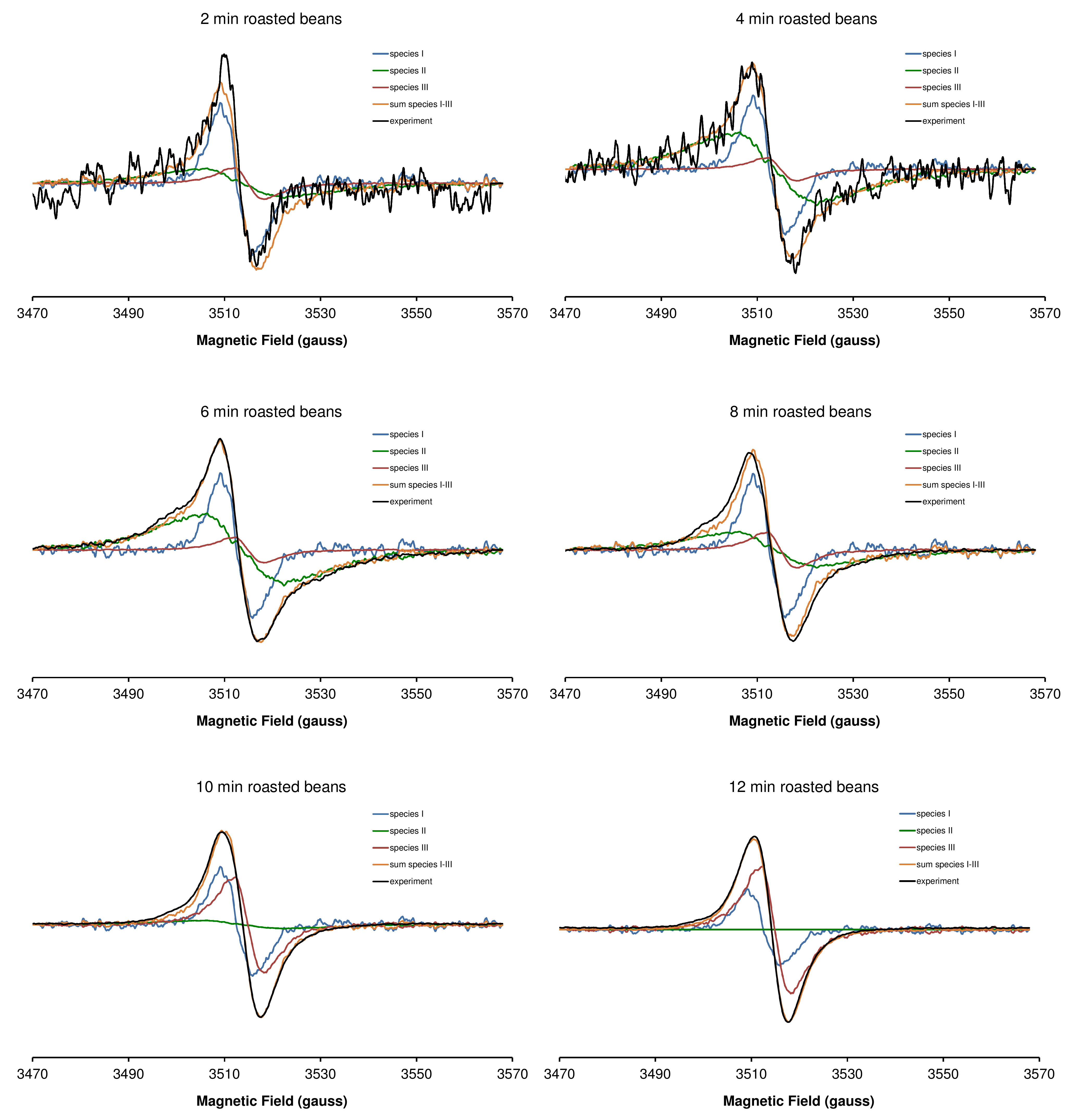

Supplement: S7 Fig — The experimental spectrum is shown in black and the reconstituted spectrum (weighted summation of species I–III) is shown in orange. The weightings are shown as a function of roasting time in Fig 2F (main text). The vertical scale of displayed spectra is arbitrary in each instance. (TIFF) [file pone.0122834.s007.tiff]

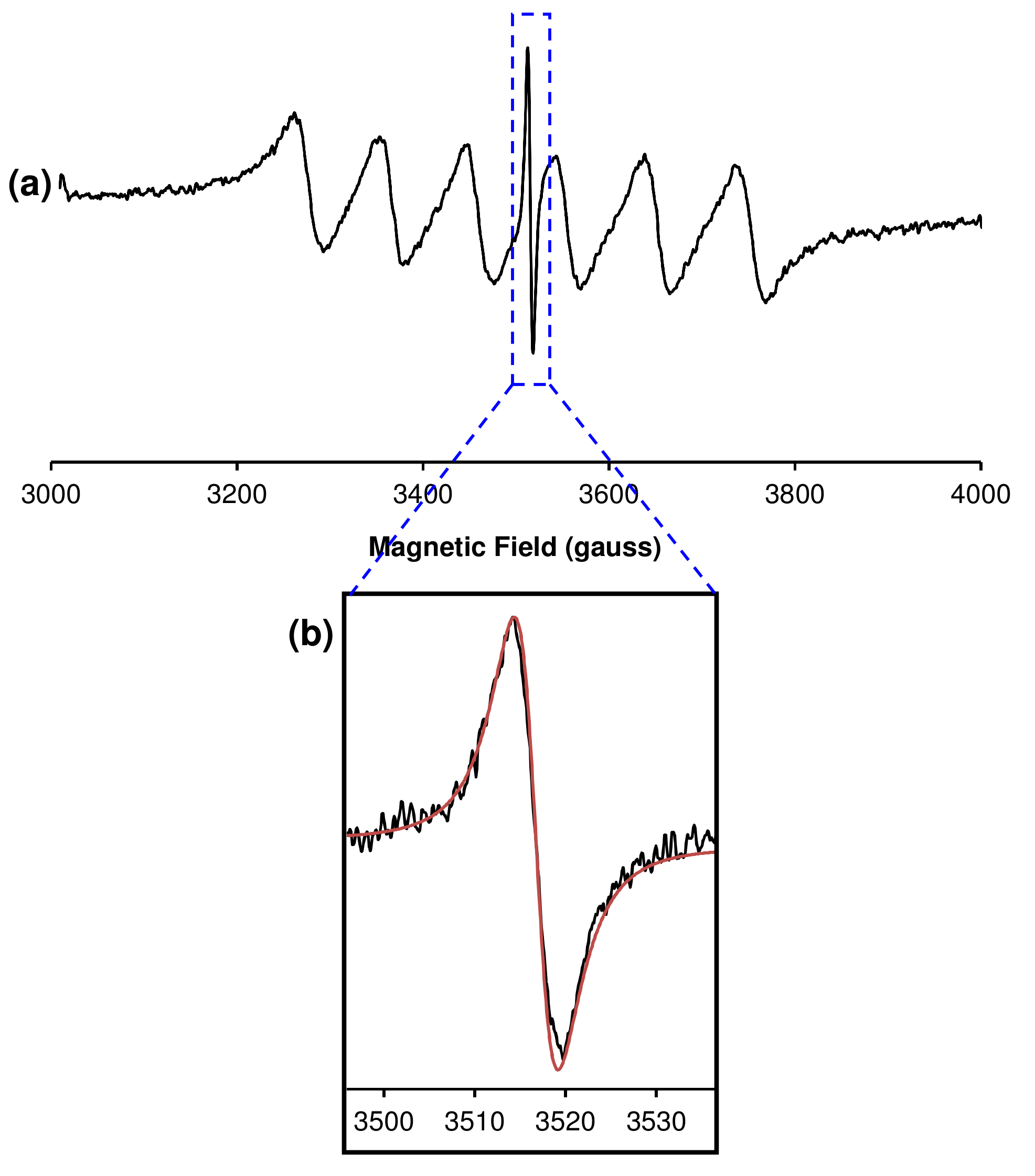

Supplement: S8 Fig — (a) Wide scan of the Mn2+ and radical signals. Microwave frequency, 9.866 GHz microwave power, 5 mW; modulation amplitude, 4 G; receiver time constant, 82 ms; receiver gain, 85 dB; sweep rate, 6.67 G/s; averages, 250. (b) Narrow scan of the radical signal, with a simulation overlaid in red. Microwave power, 10 mW; modulation amplitude, 4 G; sweep rate, 4 G/s; averages, 40. Simulation parameters are given in Table 1 in the main text. (TIFF) [file pone.0122834.s008.tiff]

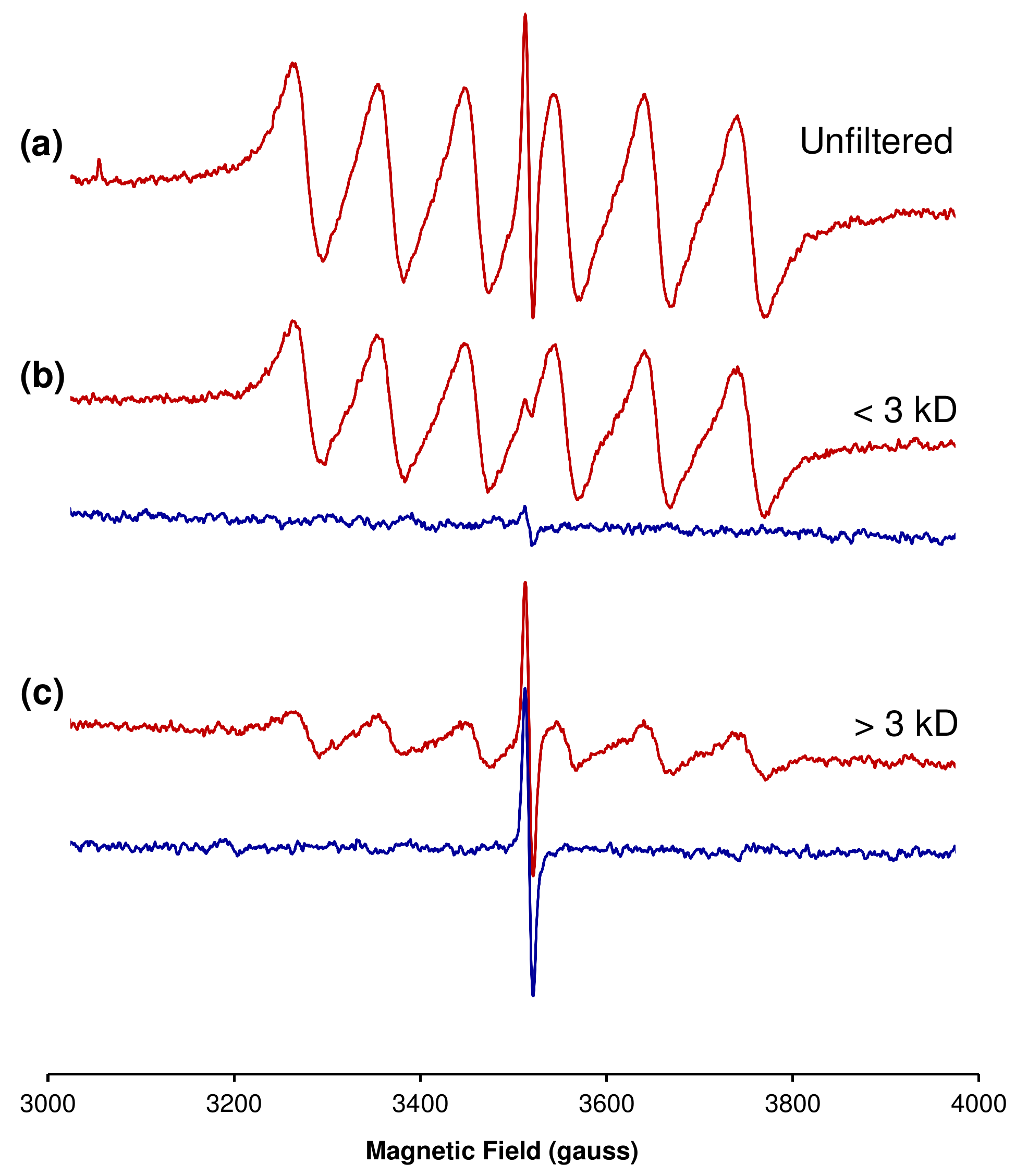

Supplement: S9 Fig — Due to its short electron spin lattice relaxation time, the Mn(DTPA) complex is effectively EPR-silent at room temperature. Hence, the complete sequestration of Mn2+ from the brew (eg. from complexes with melanoidins, polyphenols or from adventitious ions in solution) was confirmed by the disappearance of the six-line signature. (a) untreated brew; (b) filtrate from the first pass through the 3K MWCO membrane, representing the species < 3 kDa at their native concentration; (c) retentate reconstituted to the initial volume of brew, representing species > 3 kD at their native concentration. Results are representative of a single preparation of 12 min roasted beans brewed at 0.175 g/mL for 5 min at 92°C. Microwave frequency 9.860 GHz microwave power, 50 mW; modulation amplitude, 10 G; receiver time constant, 327 ms; receiver gain, 82 dB; sweep rate, 6.67 G/s; number of averages, 4. (TIFF) [file pone.0122834.s009.tiff]

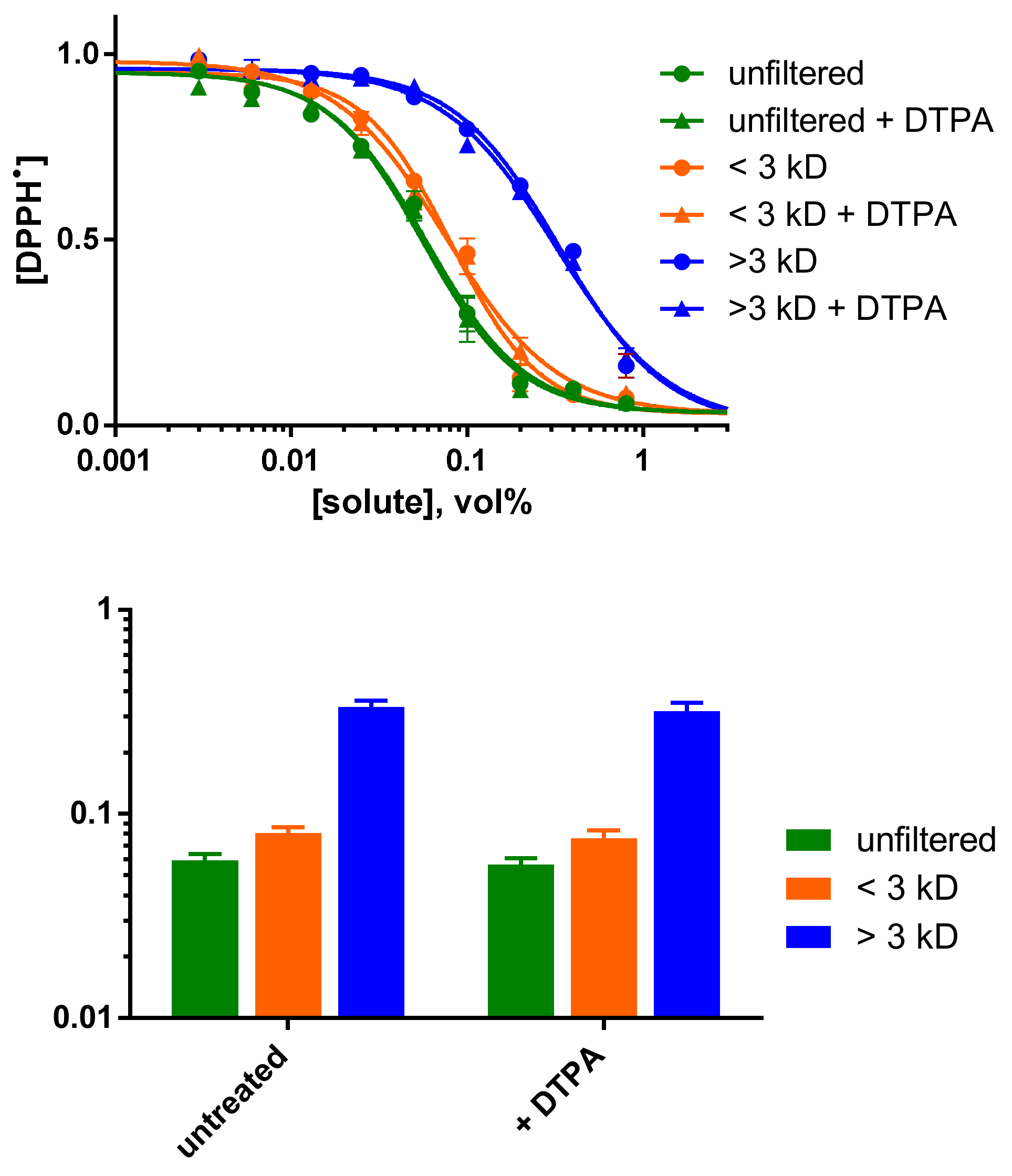

Supplement: S10 Fig — The EC50 does not change, indicating coffee metal-polyphenol and metal-melanoidin complexes do not contribute to antiradical activity. (TIFF) [file pone.0122834.s010.tiff]

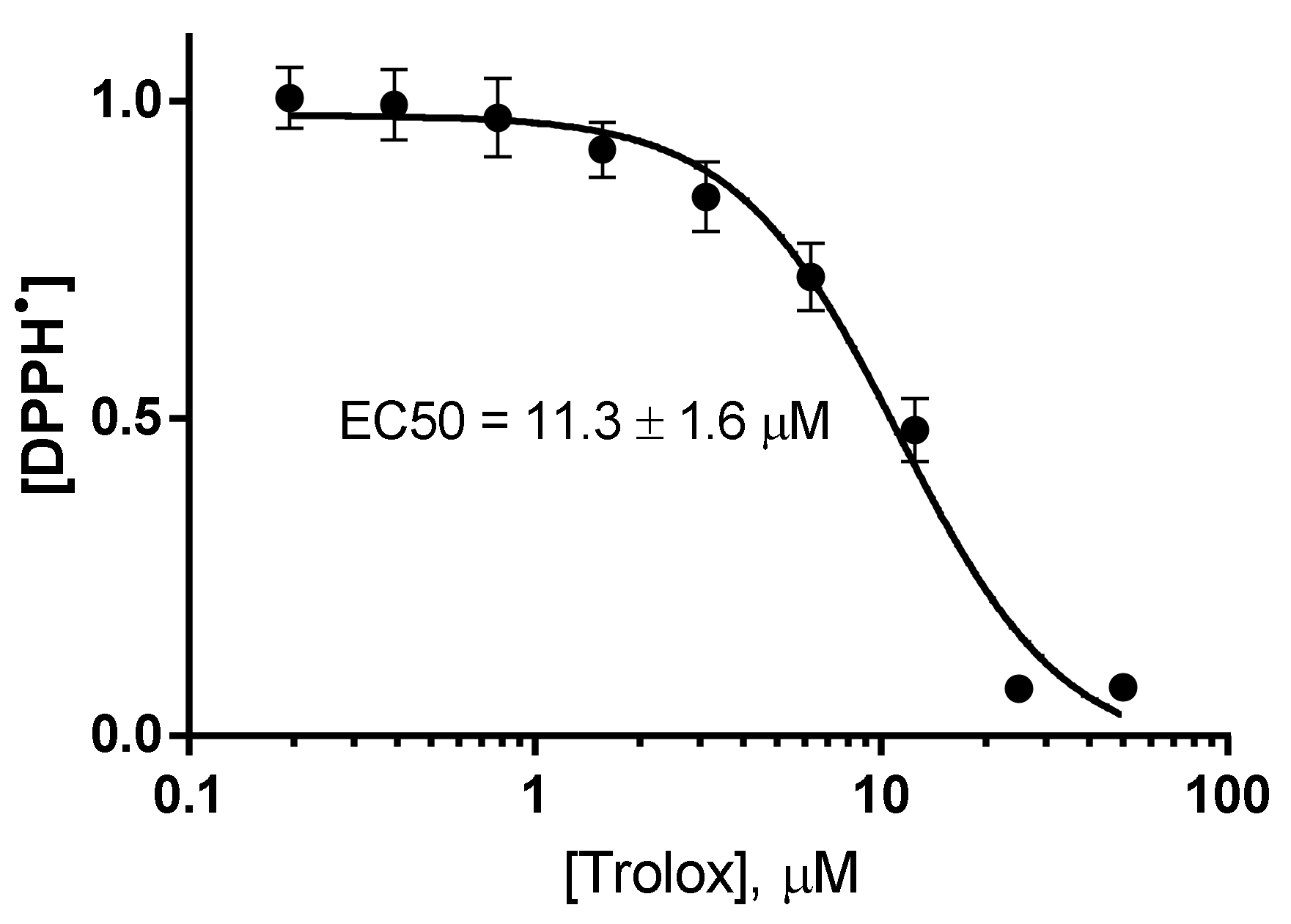

Supplement: S11 Fig — Data show the mean and SEM of 4 independent experiments. (TIFF) [file pone.0122834.s011.tiff]

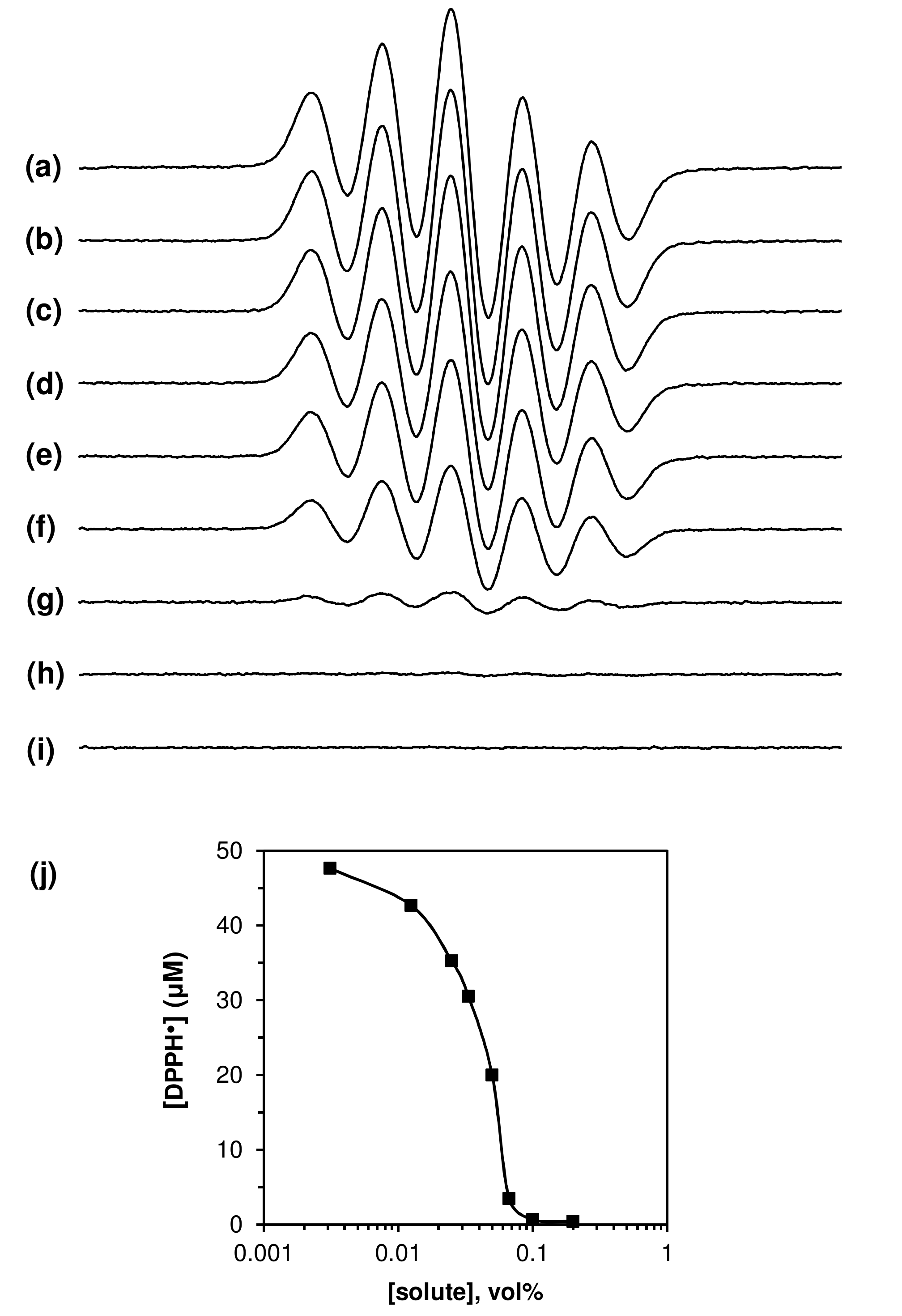

Supplement: S12 Fig — Room-temperature CW-EPR spectra of 1:1 MeOH/water solutions containing (a) 50 μM DPPH• alone, or 50 μM DPPH• with brewed coffee diluted in water by a factor of (b) 32000, (c) 8000, (d) 4000, (e) 3000, (f) 2000, (g) 1500, (h) 1000, or (i) 500. (j) Intensity of the DPPH• EPR signal shown in spectra b–i, normalized to the intensity of the 50 μM DPPH solution in spectrum a. Data represent results of a single assay performed on one brew and are therefore only qualitative. Experimental conditions: microwave frequency, 9.866 GHz; microwave power, 10 mW; magnetic field modulation amplitude, 4 G; field modulation frequency, 100 kHz; receiver time constant, 82 ms; receiver gain, 70 dB; sweep rate, 4 G/s; averages, 4. (TIFF) [file pone.0122834.s012.tiff]
