# Supplementary material for: Stable Radical Content and Anti-Radical Activity of Roasted Arabica Coffee: From In-Tact Bean to Coffee Brew
Source: PLoS One. 2015 Apr 9;10(4):e0122834. doi: 10.1371/journal.pone.0122834 (PMC4391752; doi:10.1371/journal.pone.0122834)
Supplement: S1 Table — Data represent the mean and SEM obtained from three brews prepared and assayed independently. (DOCX) [file pone.0122834.s013.docx]

| \| EC50, vol% \| unfiltered \| < 3kD \| > 3kD \| \| --- \| --- \| --- \| --- \| \| untreated \| 0.059 ± 0.004 \| 0.081 ± 0.005 \| 0.334 ± 0.025 \| \| + PVPP \| 0.170 ± 0.018 \| 0.313 ± 0.032 \| 0.742 ± 0.073 \| \| + DTPA \| 0.056 ± 0.004 \| 0.076 ± 0.007 \| 0.320 ± 0.030 \| |
| --- | --- | --- | --- | --- | --- | --- | --- | --- | --- | --- | --- | --- | --- | --- | --- | --- |
